# The Symptoms and Treatment of Outgrowths from the Back of the Tongue

**Published:** 1890-06

**Authors:** Barclay J. Baron

**Affiliations:** Physician in Charge of the Throat Department of the Bristol General Hospital


### THE SYMPTOMS AND TREATMENT OF OUTGROWTHS FROM THE BACK OF THE TONGUE.

BY

BARCLAY J. BARON, M.B. Edin.,

*Physician in charge of the Throat Department of the Bristol General Hospital.*

---

My own observation leads me to believe that in quite a fair proportion of cases of affection of the throat, whether the naso-pharynx, pharynx, or larynx be diseased, we shall find that the back of the tongue is also implicated.

If this be true—and increased experience in the examination and treatment of throat troubles enables me to feel certain that it is so—then we cannot afford to omit the examination of the region in question, if we are to accurately treat such cases.

There is no reason to doubt the homology of structure of the faucial and pharyngeal tonsils with that of the tissue which normally is found in small quantity on the extreme back of the tongue, immediately in front of the epiglottis; and it is with hypertrophy of this tissue, which has been called the "lingual tonsil," that I propose to deal.

In order to examine this region, laryngoscopy is essential, and the mirror should be placed rather higher and nearer the palate than the usual position for an examination of the larynx. Normally we see a few small irregular elevations on the back of the tongue; but the glosso-epiglottidean ligament and the fossa on either side of it are, except the epiglottis be unusually erect, quite visible, the elevations not materially encroaching on them. When, however, this tissue is hypertrophied, we find large elevations, varying in size from a pea to a large bean, with furrows on the largest ones and clefts between adjacent growths, extending all over the back of the tongue, touching the epiglottis, preventing its rising in phonation, and even so overlapping it as to entirely conceal it in extreme cases. The colour of the outgrowths varies from pink or grey, when they are not inflamed, to livid red or purple when active inflammation is present; and there is always an increase in the size of the veins of the part. The growths are often very numerous, but sometimes a few large ones cover the whole of the back of the tongue.

I have never seen them without their being accompanied by disease in some other part of the throat; usually they are found along with granular pharynx, or hypertrophy of the faucial tonsils; less commonly in cases of laryngeal catarrh, or adenoid growths of the naso-pharynx. The fact of our finding pharyngeal trouble, which accounts for *some* of the symptoms of which patients complain, is very apt to induce us to believe that they are *all* due to the pathological condition of the pharynx; and we are disappointed when we find that, whilst we do all that is right to cure what we think is the only mischief from which the patient suffers, and we see our measures to be successful, yet the case is not concluded, as various disagreeable symptoms are still present. If we then examine and treat the tongue, we shall finish the case satisfactorily; if not, we leave our patient *relieved* but not cured. On looking back to some failures to completely rid patients of throat troubles who consulted me previous to the time, since when I have never omitted to bear in mind the significance of outgrowths on the back of the tongue, I feel certain that if I could see these patients again I could cure them; and it is with a view to prevent others making the same mistakes in this direction as I have made, that I venture to write this short and imperfect paper.

The symptoms which have induced patients to consult me are very different in different cases.

Several of them have suffered from distinct interference with swallowing; *i.e.*, they have felt that some of the food lodged in the back of the throat, and that the whole of it was not cleanly swallowed. This lodgment is disposed of usually by making extraordinary efforts of deglutition, whereby it is forced into the œsophagus; in other cases patients have told me that they are obliged to hawk until the offending particle is expectorated, and then they are relieved. There is no doubt but that this unpleasant symptom is caused by food being squeezed into the spaces between adjacent growths.

In some of my cases, notably in one very severe one, in which the growths were very large, the food that lodged between the tongue and the epiglottis not only gave rise to an uncomfortable sensation, but caused a terrible feeling of impending suffocation, and the patient adopted severe measures, such as making himself retch, in order to get rid of it. There is no doubt the food crowded the epiglottis down towards the top of the larynx, and prevented its normal rise in order to get air into the lungs; also, I suppose, it set up reflex spasm, which still more tightly shut the epiglottis down. The patient, a young man, was reduced to living mainly on liquids and very soft solids, and very little even of the latter could be comfortably taken; he was very weak, practically unable to follow his occupation of a shopkeeper, and he had lost nearly 40 lbs. weight when I first saw him, and this in spite of having been treated admirably by Dr. Mackenzie, who kindly brought him to me. After removal of the outgrowths on the tongue, he got quite well, and regained nearly, if not quite, the whole of the flesh that he had lost.

Other patients have come to me complaining of "fulness in the throat," something always behind the tongue that cannot be dislodged, which leads them to "clear up" incessantly, but with no effect. This so-called "fulness" varies with the general health, and acid dyspepsia, constipation, cold damp weather, and excessive talking, as in school-teachers, will distinctly and rapidly increase it; in fact, just the conditions under which a chronically enlarged faucial tonsil will become sore and swell, and give rise to symptoms extra to those which are usually felt, so with hypertrophy of the "lingual tonsil." Within the past few days, one of my cases that was doing exceedingly well under treatment for the removal of these growths, came to me saying that she felt the back of the tongue very much enlarged and painful on movement, and I was able to see that I had to do with an intercurrent subacute inflammation, which entirely put a stop, temporarily, to all operative measures, just as it would do in the case of the faucial tonsil.

In another series of cases, the interference is with pure vocalisation: people who have had good voices, and especially high sopranos, in whom the epiglottis often becomes very erect when high notes are being sung, find that the sound appears to be crowded down into the larynx, and does not get out with the former ease; the throat becomes fatigued more easily after singing, and they do not sing with their usual pleasure, as they so soon become languid and tired. Here, I take it, the growths impinge on the front surface of the epiglottis, interfere with its assuming the erect position, and thus the exit of the sound from the larynx is impeded, and the result is poor.

Lastly, I have met with one case in which pain of a neuralgic intermittent character radiated from the throat down into the upper part of the chest and over the shoulders, which is quite gone now that the growths have been destroyed. This symptom is possibly explainable if we consider it a reflex phenomenon, similar to what we occasionally see when patients have nasal hypertrophies. Other symptoms have been described by other observers, such as intense pain, bleeding and abscess of the growth, with general fever similar to acute suppuration of the faucial tonsil, but I have no experience of them in my own practice.

As regards the treatment of this troublesome condition, it is very simple and most satisfactory, since it practically always results in cure, provided it be thoroughly carried out.

Having carefully examined all the other parts of the throat, and having treated naso-pharyngeal growths or catarrhal processes, destroyed pharyngeal granulations, removed by cutting or galvano-cautery diseased tonsils, and subdued laryngeal catarrh with suitable inhalations, sprays, brushings, &c., I proceed to apply the galvano-cautery to the tongue-growths. There is one practical point about all galvano-caustic operations in the throat; and that is, to see that the cautery is neither *white hot*, when it will cause bleeding, nor *black hot*, when it will give rise to a great deal of pain and do very little work, but of *cherry-red heat*, which is quite destructive enough, does not cause much pain, and no bleeding.

I am not in the habit of applying cocain before using the burner, but in very sensitive patients the use of a ten per cent. solution of that drug lessens pain materially, and, what is perhaps of more importance, gives them confidence and induces them to keep their heads quite steady when the circuit is closed and the burning goes on. With reference to the kind of burner to be selected, every individual case must decide this; usually I do very little, and with a small porcelain burner, at the first sitting, in order to estimate the amount of reaction that will take place. I find that it is necessary to be careful not to do too much at the first operation, and to note especially how the epiglottis behaves, since, it being impossible to entirely prevent the heat from radiating on to the anterior surface of this cartilage, where the growth that you wish to burn is nearly or quite in contact with it, you may make it swell and thus give trouble. After I am satisfied that there is no special liability to swelling and inflammation of the epiglottis and surrounding parts, I use a large flat burner, and this is to be applied to the tops of the several growths, or plunged into their substance, and thus sloughing and removal take place.

In some rare instances, where the growths are almost pedunculated, I have with advantage used the electric snare; but there is usually a good deal of irritation caused by one's efforts to accurately adjust it over the growth, and to push it down to the surface of the tongue. After each operation I enjoin rest for the voice, avoidance of hard or irritating articles of diet, and the use of ice internally and, if necessary, externally; also sucking cocain lozenges, or the use of a cocain spray, keeps down pain and inflammation. From five to seven days must elapse between the operations, and in most instances from five to ten sittings are required to clear away all the growth. When I consider that the galvano-cautery has been sufficiently applied, I prescribe a solution of chloride of zinc, 10 to 15 grains to the ounce of glycerine, as a paint for the tongue and throat.

I am convinced that the use of the electric cautery is the only proper and satisfactory method of treatment, and all attempts to reduce these growths by astringent applications only result in disappointment, because, whilst they may temporarily relieve by bracing up the throat, some slight cause, such as exposure to cold and wet, sore throat, over-use of the voice, catarrh of the stomach, &c., will bring back all the old symptoms in their former intensity. I have done the little operation in a great many cases, and although I have once or twice distinctly burnt the tip of the epiglottis, I have never had a patient come back to me before the appointed time, nor have I ever been sent for to relieve any serious condition, such as obstruction to the breathing or inability to swallow; and I consider the operation to be practically free from danger, if reasonable care be taken.

